# The Transcriptional Regulator Np20 Is the Zinc Uptake Regulator in *Pseudomonas aeruginosa*

**DOI:** 10.1371/journal.pone.0075389

**Published:** 2013-09-23

**Authors:** Matthew L. Ellison, John Matthew Farrow, Whitney Parrish, Allison S. Danell, Everett C. Pesci

**Affiliations:** 1 Department of Microbiology and Immunology, East Carolina University, Greenville, North Carolina, United State of America; 2 Department of Biology and Chemistry, Morehead State University, Morehead, Kentucky, United State of America; 3 Department of Chemistry, East Carolina University, Greenville, North Carolina, United States of America.; National Centre for Cell Science, India

## Abstract

Zinc is essential for all bacteria, but excess amounts of the metal can have toxic effects. To address this, bacteria have developed tightly regulated zinc uptake systems, such as the ZnuABC zinc transporter which is regulated by the Fur-like zinc uptake regulator (Zur). In *Pseudomonas aeruginosa*, a Zur protein has yet to be identified experimentally, however, sequence alignment revealed that the zinc-responsive transcriptional regulator Np20, encoded by *np20* (PA5499), shares high sequence identity with Zur found in other bacteria. In this study, we set out to determine whether Np20 was functioning as Zur in *P. aeruginosa*. Using RT-PCR, we determined that *np20* (hereafter known as *zur*) formed a polycistronic operon with *znuC* and *znuB*. Mutant strains, lacking the putative *znuA, znuB*, or *znuC* genes were found to grow poorly in zinc deplete conditions as compared to wild-type strain PAO1. Intracellular zinc concentrations in strain PAO-Zur (Δ*zur*) were found to be higher than those for strain PAO1, further implicating the *zur* as the zinc uptake regulator. Reporter gene fusions and real time RT-PCR revealed that transcription of *znuA* was repressed in a zinc-dependent manner in strain PAO1, however zinc-dependent transcriptional repression was alleviated in strain PAO-Zur, suggesting that the *P. aeruginosa* Zur homolog (ZurPA) directly regulates expression of *znuA*. Electrophoretic mobility shift assays also revealed that recombinant ZurPA specifically binds to the promoter region of *znuA* and does not bind in the presence of the zinc chelator *N,N*′,*N*-tetrakis(2-pyridylmethyl) ethylenediamine (TPEN). Taken together, these data support the notion that Np20 is the *P. aeruginosa* Zur, which regulates the transcription of the genes encoding the high affinity ZnuABC zinc transport system.

## Introduction

Zinc is an essential trace element required for virtually all forms of life. The importance of the metal is demonstrated by the fact that 4% to 10% of all proteins found throughout the domains of life are zinc metalloproteins [1]. Zinc serves many important functions in such proteins, including acting as a co-factor to facilitate enzymatic reactions, serving as a structural component of many ribosomal proteins and transcriptional regulators, and assisting in proper protein folding and formation. In addition, zinc has been shown to protect against harmful free radical formation which can occur through normal aerobic metabolism within a cell [2]. Accordingly, bacteria concentrate zinc to high levels within the cell to maintain these processes.

In *E. coli*, the intracellular concentration of zinc is approximately 0.2 mM, regardless of the zinc concentration in the external milieu [3]. However, even though the intracellular zinc concentrations may be 1000-higher than the extracellular environment, there is typically no free zinc ions found inside the cell [3]. This suggests that the cell possesses means to actively take in the metal and to prevent excess free zinc from accumulating within the bacterium.

Although zinc is essential, excess zinc can have deleterious effects on cells [4]. At physiological concentrations, zinc may have a protective role against free radical formation [5,6]. However, surplus zinc has been shown to induce cell toxicity by competing against other metals for protein binding [7] and exposure to high zinc concentrations has been speculated to cause protein denaturation and dysfunction [8]. In addition, zinc induced envelope stress has been shown to disrupt normal physiological processes [9]. Thus, bacteria must not only have the means to take in Zn(II), but also must repress uptake or quickly export any excess zinc ions to maintain homeostasis.

To ensure sufficient Zn(II) is available for cellular functions, many bacteria possess a high affinity active zinc uptake system known as ZnuABC. First identified in *Escherichia coli*, this ABC-type transport system is comprised of a zinc-binding periplasmic protein (ZnuA), an inner membrane permease (ZnuB), and an ATPase (ZnuC) [10]. In *E. coli*, Zn(II) uptake by the ZnuABC system is controlled by the zinc uptake regulator (Zur). Zur, a member of the Fur-like family of transcriptional regulators, has the ability to sense and respond to femtomolar changes in zinc concentration within the cell [3]. Zur has at least two zinc binding sites, one zinc binding site (C site) that allows for structural stability and one or two binding sites (M and D sites) which regulate DNA binding [11-13]. Under zinc-replete conditions, excess zinc ions interact with the M and/or D sites, which causes a conformational change that allows the regulator to bind to the *znuA* promoter and thereby repress its transcription. In zinc-deplete conditions, the M and/or D sites are unoccupied which leads to destabilization of Zur and the regulator is unable to repress *znuA* transcription, thus allowing zinc uptake [6].

Our interest in zinc homeostasis, and specifically Zur, stems from recent work with *Pseudomonas aeruginosa. P. aeruginosa* is a ubiquitous gram-negative bacterium found in numerous ecological niches. As an opportunistic pathogen, it can infect a wide range of organisms, including insects, nematodes, plants, and animals [14-18]. In humans, *P. aeruginosa* can cause debilitating disease in the immunocompromised, is a major contributor to nosocomial infections, and is the leading cause of morbidity and mortality in individuals suffering from cystic fibrosis [19]. In order to establish infections, this pathogen produces a host of virulence factors, including the phenazine, pyocyanin. In a study to identify genes involved in the production of pyocyanin, mutation of the *np20* gene which was previously shown to be a virulence determinant [20], caused deficiency in both pyocyanin and the cell-to-cell signal 2-heptyl-3-hydroxy-4-quinolone (the *Pseudomonas* Quinolone Signal or PQS) production [21]. Others have reported that this regulator is required for production of pyocyanin [22], and that *np20* mutant strains are avirulent in both nematode [15] and mouse infection models [14]. Recently, Np20 was shown to regulate the expression of *dksA2*, a paralogue of global transcriptional regulator *dksA*, in a zinc-dependent manner [23]. The controlled expression of *dksA2* by Np20 allowed *P. aeruginosa* to regulate genes controlled by the zinc-dependent DksA transcriptional regulator in low zinc conditions. Thus, Np20 appears to be involved in the expression of numerous *P. aeruginosa* genes and is responsive to zinc concentration. Despite multiple studies describing its effects, the role of Np20 in regulating zinc homeostasis has not been investigated experimentally. Likewise, no zinc uptake regulator has been identified in *P. aeruginosa*. Based on these observations, we hypothesized that Np20 regulates zinc homeostasis in *P. aeruginosa*. In this study, we present experimental evidence that Np20 serves as a regulator of zinc uptake and hereafter refer to it as Zur_PA_.

## Results

### Zur_PA_ is Highly Similar to Zur Found in Other Bacteria

**Figure 1:**
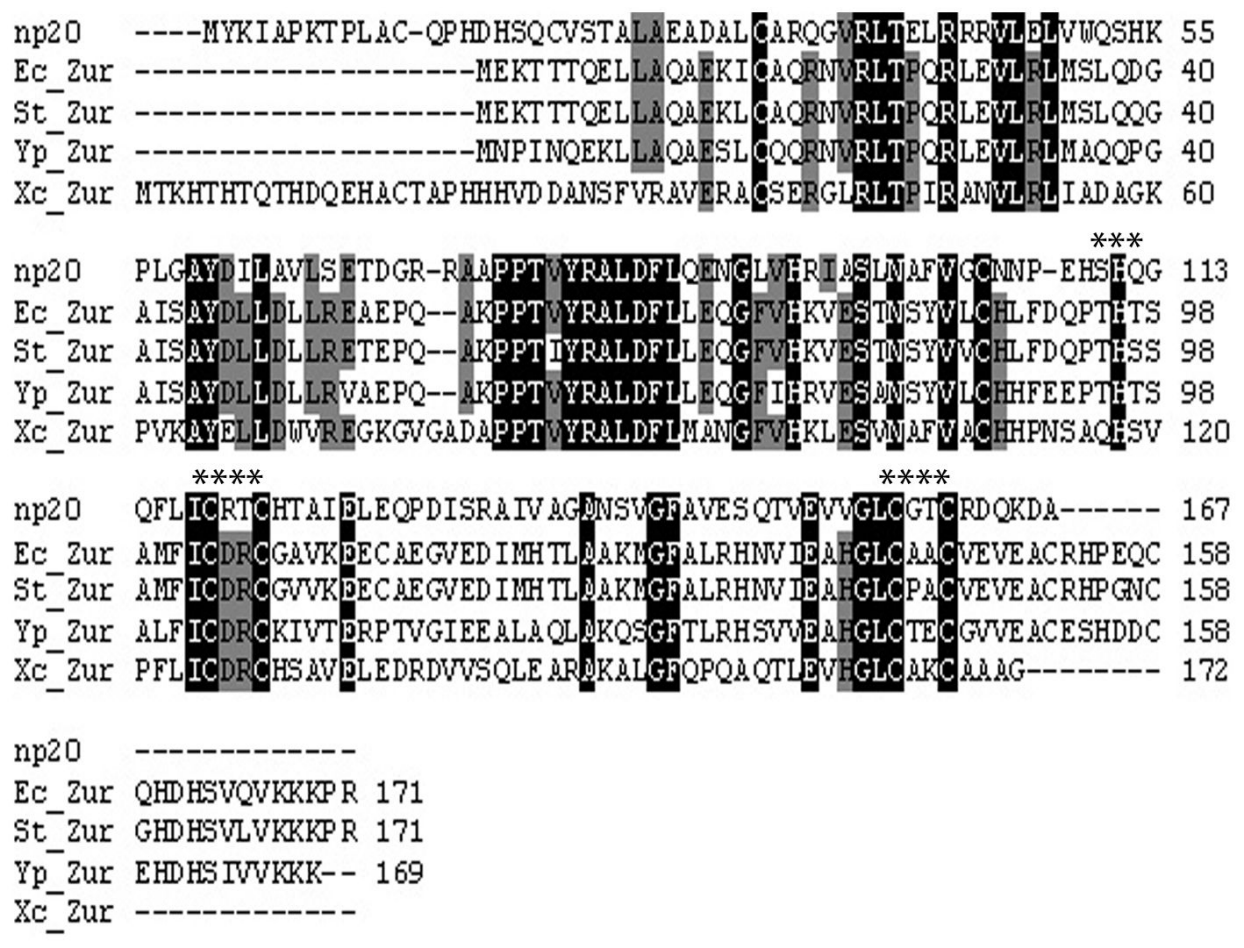
Sequence alignment of Zur_PA_ with other bacterial Zur proteins. The protein sequence of Zur_PA_ (Np20) was aligned with Zur proteins from the following bacteria: *E. coli* (Ec_Zur); *S* *. typhimurium* (St_Zur); *Y. pestis* (Yp_Zur); *X. campestris* (Xc_Zur) using ClustalW. Completely conserved residues are indicated by the black shading. Partially conserved residues are indicated by grey shading. Asterisks indicate the C and M zinc binding sites.

If the protein encoded by *np20* is the *P. aeruginosa* Zur, it would be expected to share sequence similarity with Zur proteins found in other bacteria. To address this, we aligned and compared the amino acid sequence of the putative Zur_PA_ with that of Zur proteins previously characterized in other bacteria (Figure 1). As can be seen in Figure 1, Zur_PA_ has high sequence identity with other Zur proteins. It has greater than 40% identity with the Zur protein from the gram-negative bacteria examined and 45% similarity (31% identity) with FurB from *M. tuberculosis* (data not shown). Zur_PA_ also shares several regions of conserved sequences with the other Zur proteins (indicated by the black shading). In addition to containing the first Zur specific sequence, a highly conserved putative DNA-binding region from amino acids 76 to 92, Zur_PA_ also has two conserved zinc binding domains. The double cysteine-X-X-cysteine motif found in C_118_XXC_121_-X_36_-C_158_XXC_161_ is completely conserved in all characterized Zur proteins and is highly conserved throughout the Fur-like protein family [13]. This site corresponds to the structural zinc binding C-site described in other Zur proteins. The other highly conserved site, which includes two histidine residues in the H_109_ SH_111_ motif and the highly conserved C_104_ is indicative of the M-site, which is a zinc sensing site [13]. Zinc interaction with the M-site and portions of the DNA-binding site alters Zur confirmation and allows for DNA binding. The occurrence of the putative C and M zinc binding sites in Zur_PA_ and the high overall similarity between Zur_PA_ and Zur proteins from other bacteria strongly suggested that the protein encoded by *np20* is the *P. aeruginosa* Zur (Zur_PA_).

### *zur* is in an Operon Which Encodes Components of the Putative ZnuABC System

**Figure 2:**
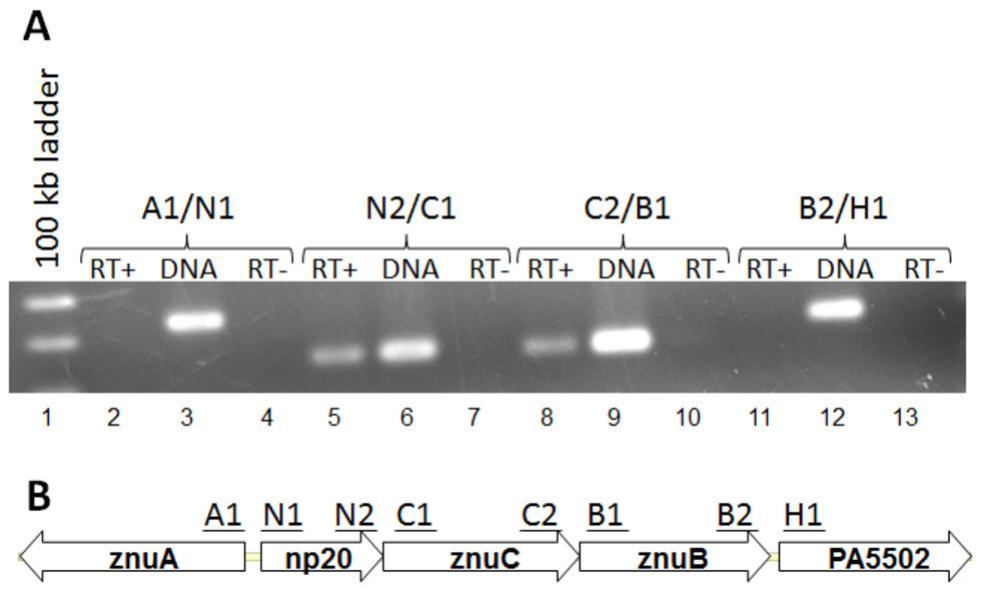
*zur* is encoded in an operon with *znuC* and *znuB*. (A) RT-PCR was performed on total RNA isolated from *P. aeruginosa* strain PA01 using the indicated primer pairs (see Table S2). Reverse Transcriptase was added to the reaction in lanes 2, 5, 8, and 11(RT+) but omitted from reactions in lanes 4, 7, 10, and 13 (RT-). Genomic DNA was amplified using the indicated primers as a positive control in lanes 3, 6, 9, and 12 (DNA); (B) Schematic representation of the *zur* (np20) operon with relative location of the indicated primers used for RT-PCR.

We next set out to determine the genetic organization of the operon containing the *zur* gene. The annotation provided at www.Pseudomonas.com suggested that *zur* was the first gene in a three gene operon. The genes predicted to be transcribed within the *zur* operon include homologs of the *E. coli znuC* (61% identity) and *znuB* (60% identity). Using reverse transcription-PCR, we found that primers designed to span the intergenic regions between *zur* and the *znuC* homolog, as well as *znuC* and *znuB* homolog each generated a PCR product (Figure 2A). This indicated that these genes were transcribed on the same mRNA. Interestingly, primers spanning the 42 bp intergenic region between the putative *znuB* and a hypothetical gene, PA5502, did not give a product, suggesting that gene PA5502 is not part of the *zur* operon, despite its proximal location to the *zur-znuC-znuB* operon. As expected, primers spanning the 70 bp intergenic region between *zur* and the divergently transcribed gene PA5498, which shares 60% sequence similarity with the *E. coli znuA* (identified as *znuA* in Figure 2A), did not generate a product. These data suggested that *zur* forms an operon with two of the three genes which encode components of a probable ZnuABC zinc uptake system, further implicating the involvement of Zur_PA_ with the zinc uptake system. A schematic representation of the Zur operon is shown in Figure 2B.

### Loss of the Putative ZnuA, ZnuB, and ZnuC Effects Growth in Low Zinc Conditions

**Figure 3:**
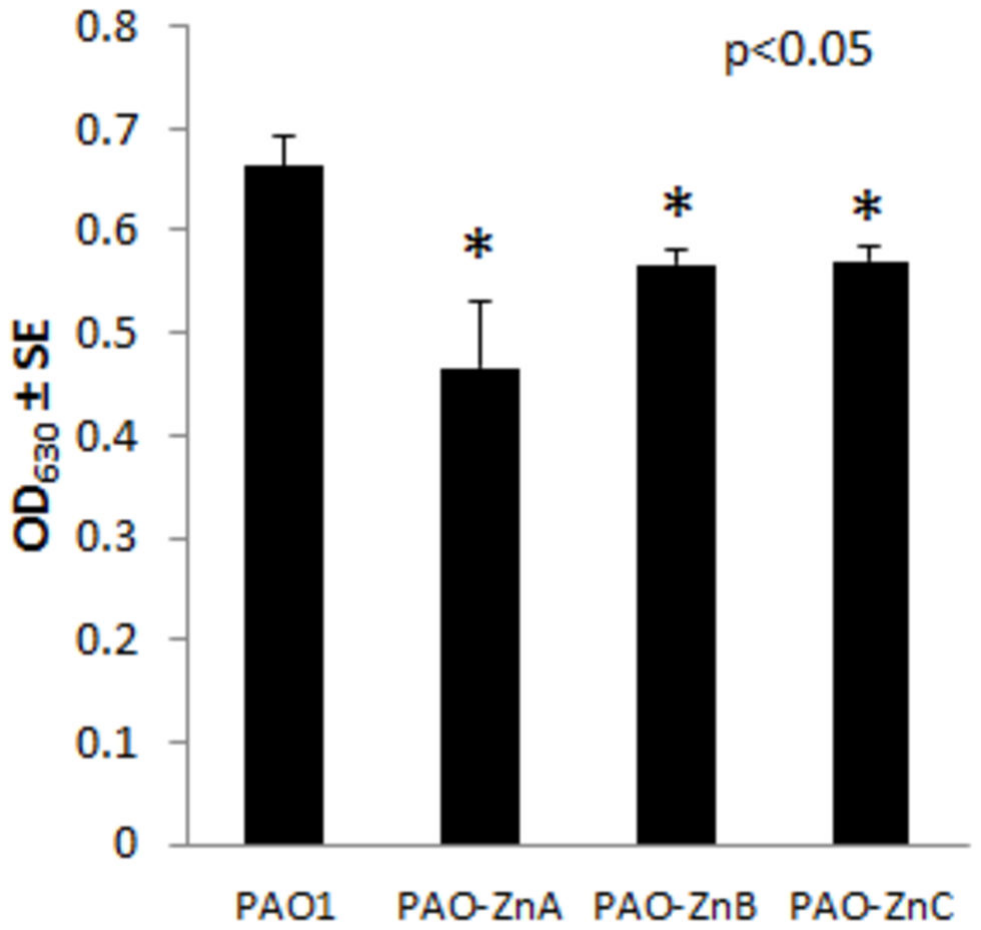
Putative *znuA, znuB*, and *znuC* genes are required for wild-type level growth in low zinc conditions. Overnight cultures were diluted in LB media containing 0.5 mM EDTA and grown overnight at 37°C for 18 hours with rotary aeration ≥ 220 rpm. Growth was assessed spectrophotometrically at absorbance 630 nm. The results are given as the mean of three independent biological replicates. Asterisks indicate statistically significant less growth as compared to wild-type strain PAO1.

The similarity of the genes transcribed in the *P. aeruginosa* zur operon to genes in *E. coli* that encode components of the ZnuABC transport system strongly suggest the *P. aeruginosa* homologs would have a similar function. However, many ATPase metal transport systems share high sequence similarities and yet do not transport the same metals. In order to test if the *P. aeruginosa* putative *znuA, znuB*, and *znuC* genes may be involved in zinc uptake, we constructed deletion mutants of the putative genes PAO-ZnA (Δ*znuA*), PAO-ZnB (Δ*znuB*), and PAO-ZnC (Δ*znuC*), then grew the strains in zinc deplete conditions. As seen in Figure 3, the inability to express the putative *znuA, znuB*, or *znuC* genes resulted in decreased growth at 18 hours as compared wild-type strain PAO1 when the strains were grown in LB media supplemented with 0.5 mM EDTA. The growth defects were not severe (29.6% less growth in strain PAO-ZnA, 14.6% less growth in strain PAO-ZnB, and 14.1% less growth in strain PAO-ZnC as compared to wild-type strain PAO1), yet they were found to be statistically significant. The ability to grow at all in low zinc can be attributed to *P. aeruginosa* possessing low affinity zinc permeases (such as HmtA) that can uptake the metal in the absence of a functional ZnuABC transporter. Because these mutants have decreased growth in low zinc conditions compared to wild-type strain PAO1, and the genes have high sequence similarity to the corresponding genes in *E. coli*, these data support the idea that these genes encode the ZnuABC transporter.

To confirm that the mutation of the putative *znuA, znuB*, or *znuC* genes did not cause a general growth defect, we analyzed the growth kinetics of strains PAO1, PAO-ZnA, PAO-ZnB, and PAO-ZnC grown in LB media (see Figure S1). None of the mutant strains showed growth defects when grown in zinc replete conditions.

### Loss of Np20 (Zur) Results in an Accumulation of Intracellular Zinc

**Figure 4:**
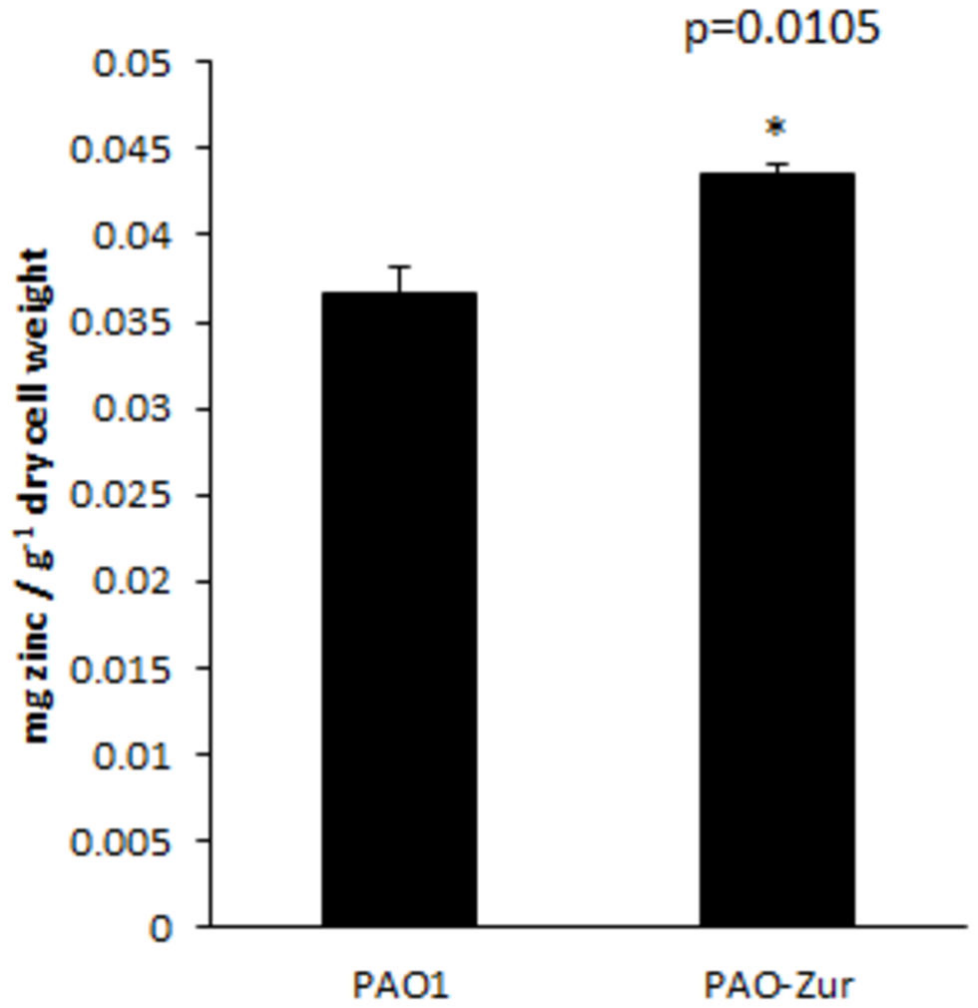
Loss of Zur results in intracellular zinc accumulation. Intracellular zinc content in strains PAO1 and PAO-Zur after growth in high zinc medium as measured by ICP-AES. The data presented are the mean ± SD of at least four replicates. The asterisk indicates a significant difference between intracellular zinc in stain PAO1 versus PAO-Zur.

If Np20 is the *P. aeruginosa* Zur, then it should repress zinc uptake in high zinc conditions. Thus, loss of the protein should result in a higher accumulation of intracellular zinc compared to a wild-type strain. To test this hypothesis, we grew strains PAO1 and PAO-Zur in high zinc conditions and then determined the intracellular zinc content by ICP-AES. Our results, shown in Figure 4, show that loss of Zur in strain PAO-Zur results in a 15.6% increase of intracellular zinc compared to wild-type strain PAO1 (0.0435±0.006 mg zinc / g^-1^ dry PAO-Zur cell weight versus 0.0367±0.002 mg zinc / g^-1^ dry PAO1 cell weight). Zinc accumulation inside the PAO-Zur strain did not reach toxic levels, more than likely a result of the activation of zinc efflux systems. However, the results support the notion that Np20 is the *P. aeruginosa* Zur (Zur_PA_) and controls zinc uptake in this bacterium*.*

### Transcription of znuA is Under the Control of Zur_PA_ in a Zinc-Dependent Manner

**Figure 5:**
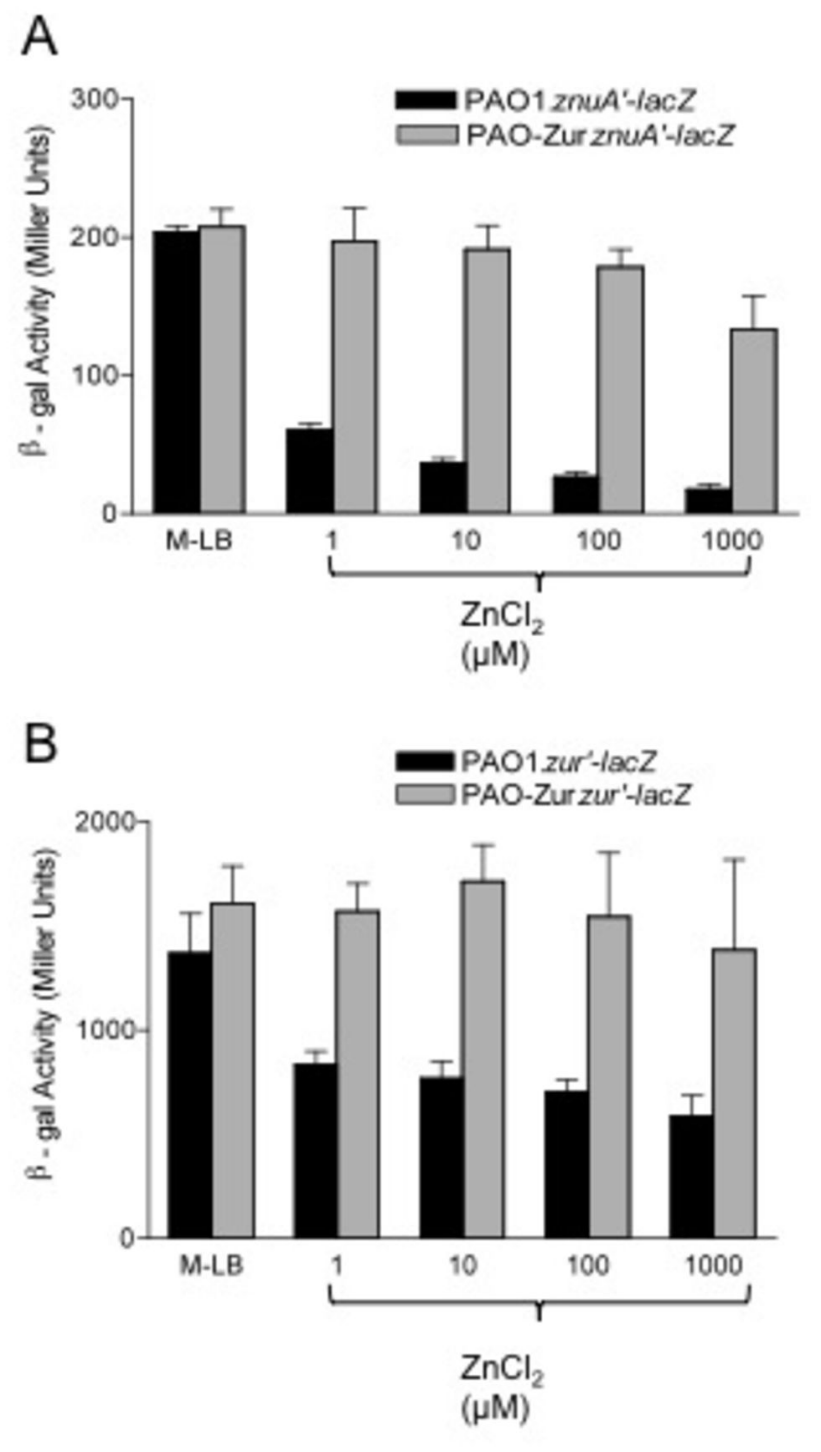
Zur_PA_ regulates transcription of *znuA* and itself. (a) β-gal assays showing strains PAO1 (black bar) and PAO-Zur (Δ*zur*) (grey bar) containing a chromosomal *znuA*’*-lacZ* transcriptional fusion grown in increasing concentrations of ZnCl_2_; (B) β-gal assays showing strains PAO1(black bar), PAO-Zur (Δ*zur*) and (grey bar) containing a chromosomal *zur*’*-lacZ* transcriptional fusion grown in increasing concentrations of ZnCl_2_. The results are presented in Miller Units ± SD as the average of at least three independent experiments.

To determine the role of Zur_PA_ on *znuA* transcription, we constructed two reporter strains, PAO1.*znuA*’*-lacZ* and PAO-Zur.*znuA*’*-lacZ*, which contain a *znuA*’-*lacZ* transcriptional fusion on their chromosomes in a neutral, non-mutational site. The strains were grown to mid-log phase in modified media supplemented with increasing ZnCl_2_ concentrations, and β-gal activity was measured. As seen in Figure 5A, *znuA* transcription was virtually the same for both strains when grown in low zinc media (204 ± 4 vs. 208 ± 13 Miller units). However, the addition of 1µM ZnCl_2_ greatly decreased *znuA*’-*lacZ* activity in strain PAO1.*znuA*’*-lacZ* to 61 ± 5 Miller units while it had virtually no effect on *znuA*’-*lacZ* activity in strain PAO-Zur.*znuA*’*-lacZ* (197 ±24) (Figure 5A). Transcription of *znuA* in strain PAO1.*znuA*’*-lacZ* decreased step-wise as the ZnCl_2_ concentrations increased, dropping by greater than 10-fold from M-LB (204 ± 4 to 18 ± 4 Miller units) to M-LB plus 1 mM ZnCl_2_ (Figure 5A). Transcription of *znuA* in strain PAO-Zur.*znuA*’*-lacZ* remained relatively unchanged before dropping 35% when ZnCl_2_ concentration reached 1 mM, indicating that Zur_PA_ is required for a response to zinc. Expression of Zur_PA_ on plasmid pZur in strain PAO-Zur.*znuA*’*-lacZ* when the strain was grown in ≥100 µM zinc resulted in a drop in Miller units to 15.7 ± 7, which mirrors the Miller Units for strain PAO1.*znuA*’*-lacZ* grown under the same conditions (data not shown)*.*

To confirm the β-galactosidase assay results, we performed real time RT-PCR. We compared expression levels of *znuA* in strain PAO1 and PAO-Zur grown in M-LB supplemented with 1 mM ZnCl_2_. Using the house keeping gene *rplU* to standardize the comparison, we found that *znuA* expression was 5.7 ±1.8 fold higher in strain PAO-Zur than in strain PAO1 when grown in high zinc (data not shown). The fold difference is similar to the 7.5 fold difference in Miller Units between observed between the *znuA*’*-lacZ* reporter strains. Collectively, these data support the hypothesis that Zur_PA_ repressed *znuA* transcription in a zinc dependent manner. They also further suggest that ZnuABC was involved in Zn(II) homeostasis.

### Zur_PA_ Autoregulates its Own Transcription

Because the intergenic region between *znuA* and *zur* is only 70 bp, we speculated that Zur_PA_ may also regulate its own transcription. To test this, we constructed a chromosomal *zur*’-*lacZ* transcriptional fusion reporter, introduced it into a neutral site in strains PAO1 and PAO-Zur, and measured β-galactosidase activity. As with the *znuA*’*-lacZ* fusion grown in M-LB, transcription of *zur* in both strains was similar (1368±191 Miller units vs. 1607±182 Miller units; Figure 5B). However, when the strains were grown in the presence of increasing concentrations of ZnCl_2_, *zur* transcription was repressed in a step-wise manner in strain PAO1, dropping to 589±96 Miller units when grown in 1000 µM ZnCl_2_ (Figure 5B). Transcription of *zur* remained virtually unchanged in strain PAO-Zur.*zur*’*-lacZ* regardless of the zinc concentration in the media. Overexpression of Zur_PA_ on plasmid pZur in PAO-Zur.*zur*’*-lacZ* in the presence of excess zinc resulted in almost full complementation of *zur* repression (798±180 Miller units in PAO-Zur.*zur*’*-lacZ* (pZur) versus 705±58 Miller units in strain PAO1. *zur’-lacZ*) when both strains were grown in M-LB plus 100 µM ZnCl_2_ (data not shown). These data support the notion that Zur_PA_ represses transcription of its own operon in response to increases in zinc concentration.

### Zur_PA_ Directly Interacts with the *znuA* Promoter

**Figure 6:**
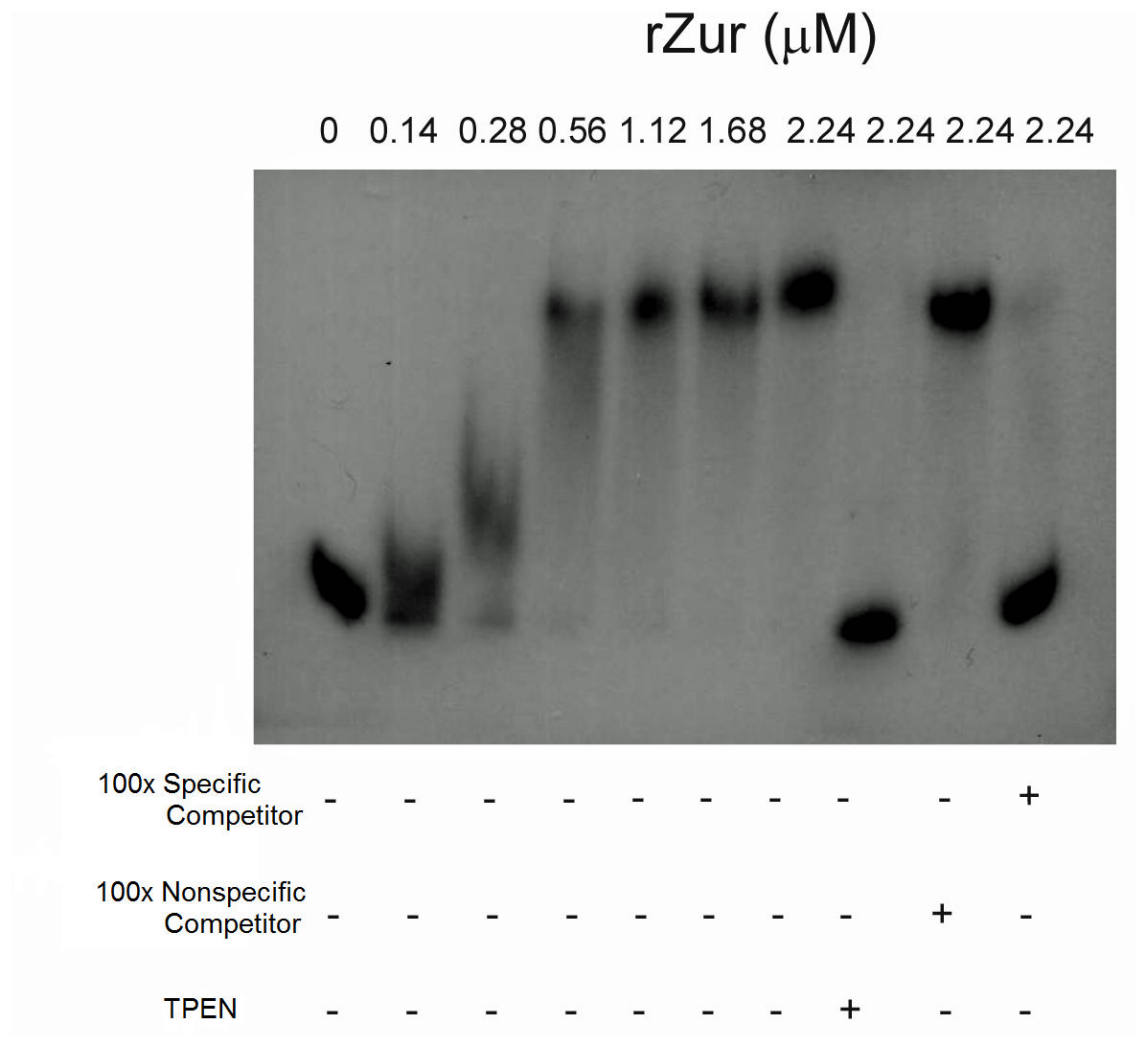
*P. aeruginosa* Zur binds the *znuA* promoter in a zinc dependent-manner. Radiolabeled *znuA* promoter fragment was incubated with increasing concentrations of recombinant *P. aeruginosa* Zur (rZur) as indicated above the figure. The plus (+) or minus (-) symbols below the lanes indicate whether the reaction contained a 100-fold excess of unlabeled *znuA* promoter fragment (specific competitor), unlabeled internal *znuC* DNA fragment (nonspecific competitor), or 5 µM TPEN (zinc chelator).

The above data suggested that Zur_PA_ represses *znuA* transcription in a zinc-dependent manner; however it was not clear if it does so directly. To test whether Zur_PA_ directly or indirectly regulates the expression of *znuA*, we performed electrophoretic mobility shift assays (EMSA). Recombinant Strep-tagged Zur_PA_ (rZur) was expressed and purified from *E. coli* strain BL21(DE3) (prZur) and used in EMSA experiments. *P. aeruginosa* rZur bound specifically to the 198 bp fragment which contains the *znuA* promoter in intergenic region spanning between *znuA* and *zur* (Figure 6). The ability of rZur to bind the promoter depended on the availability of free zinc in the buffer reaction. The addition of TPEN, a zinc specific chelator, to the binding reaction rendered rZur unable to bind to the promoter. These findings show that Zn^2+^ can modulate the binding activity of *P. aeruginosa* Zur to DNA and further support the notion that Zur_PA_ serves as a zinc-responsive transcriptional repressor of *znuA* and *zur-znuCB.*

## Discussion

Most living things require zinc for survival, however excess amounts of this trace element can be toxic. For this reason, most bacteria have developed systems for both zinc acquisition and detoxification. In addition to systems for zinc uptake and efflux, many bacteria have repressors that limit zinc import as the intracellular zinc quota is reached. In this study, we have presented data which support the notion that the transcriptional regulator encoded by gene *np20* in *P. aeruginosa* acts as a Zur protein by controlling zinc uptake through the zinc specific ABC-transport system ZnuABC. Zur_PA_ is zinc responsive and represses transcription of both *znuA* and the *zur-znuC-znuB* operon in a zinc dependent manner. Loss of Zur_PA_ results in constitutive transcription of *znuA* and *zur*, regardless of the zinc concentration in the growth media. Loss of Zur also results in the accumulation of intracellular zinc in *P. aeruginosa*. One might expect the loss of Zur to be lethal due to uncontrolled zinc uptake through ZnuABC, but we observed no toxic effects at zinc concentrations at 100 µM and only slight effects at extremely high zinc concentrations (data not shown). The reason for this lies in the fact that *P. aeruginosa* possesses several zinc efflux systems. Two such transporters have been characterized in *P. aeruginosa* to date (HmtA and CzcABC) and several other potential zinc transport systems exist on the genome [24,25]. Our growth data for strain PAO-Zur suggests that loss of Zur does not have deleterious effects as were reported with a CzcA knockout strain (20). Thus, the combined interplay between Zur and zinc efflux systems could explain the minor growth phenotypes observed in strain PAO-Zur. Interestingly, strains PAO-ZnA, PAO-ZnB, and PAO-ZnC seemed to grow slightly better than the wild-type strain PAO1 on LB plates supplemented with 10 mM ZnCl_2_ (data not shown), suggesting that eliminating zinc uptake through ZnuABC offered a protective effect against zinc toxicity. The fact that strains PAO-ZnA, PAO-ZnB, and PAO-ZnC grew well in any media suggests that *P. aeruginosa* can acquire sufficient zinc to sustain life through other means yet to be characterized.

In addition to regulating ZnuABC, studies have begun to expand the Zur regulon far beyond initial expectation. Zur has been shown to regulate a plethora of genes in bacteria, including, zinc acquisition genes [26,27], genes associated with zinc efflux [28], transcriptional regulators [23,29,30], biosynthetic genes responsible for coelibactin production in *Streptomyces coelicolor* [31], and several ribosomal proteins [13,20,32,33]. While the link to genes involved in regulating zinc homeostasis is expected, closer examination of the role that Zur plays in ribosomal protein expression revealed a plausible explanation for a zinc connection. Ribosomal proteins are typically classified into two classes based on their usage of zinc as a cofactor [34]. Zinc-utilizing ribosomal proteins contain two CXXC motifs, similar to the C site found in Zur. Not surprisingly, Zur regulated ribosomal proteins tend to lack zinc binding motifs. Under zinc-replete conditions, Zur represses production of these proteins, leading to preferential production of zinc-utilizing ribosomal proteins. Under zinc-deplete conditions, non-zinc binding proteins make up a majority of the ribosomes [32,34,35]. Thus, Zur can modulate the expression of the proper class of ribosomal proteins and lead to continual protein synthesis in cells in response to zinc availability. In fast growing bacteria, where each cell may contain up to 70,000 ribosomes [36], this tight regulation is essential to maintain population growth.

The link between Zur and virulence associated transcriptional regulators has sparked a growing interest in recent years. Zur positively regulates the hypersensitivity response and pathogenicity (hrp) transcriptional regulator *hrpX* in *Xanthomonas campestris* [29]. HrpX, in turn, regulates expression of the *hrpA* to *hrpF*, which is required for full virulence in this plant pathogen. A complete Zur – ZnuABC system is required for production of virulence factors and for full virulence in several other pathogens [37-43]. In *P. aeruginosa*, Zur_PA_ mutants are avirulent in neutropenic mice and nematode infection models and have been reported to produce less PQS [21]. Interestingly, we did not see a difference in PQS production between the wild-type strain PAO1 or mutant strains PAO-Zur, PAO-ZnA, PAO-ZnB, or PAO-ZnC (data not shown). Loss of *znuB* caused *P. aeruginosa* to lose cyanide based *Caenorhabditis elegans* killing [15]. It is somewhat interesting that despite the links to virulence in some bacteria, other pathogens have no requirement for a fully functional Zur – ZnuABC for virulence [44].

Although not as well studied as Fur and iron homeostasis, the expanding knowledge of the Fur-like regulator Zur and the ZnuABC zinc import system suggests that this system is important in numerous physiological processes beyond zinc homeostasis. While parts of the zinc homeostasis system have been identified, the complete system for maintaining the intracellular zinc quota is not well understood. This gap in knowledge along with the link between virulence and zinc homeostasis in *P. aeruginosa* suggests that this field of study shows great promise for helping to understand the role of metals during infection.

## Materials and Methods

### Bacterial Strains, Plasmids, and Growth Conditions

Bacterial strains and plasmids used in this study are listed in Table S1. *P. aeruginosa* strains were maintained at -70°C in 10% skim milk (Becton Dickinson) and *E. coli* strains were maintained in 15% glycerol. All cultures were freshly plated for each experiment. Bacteria were cultured in Luria-Bertani (LB) media [45] or zinc deplete modified-LB (M-LB) as noted below. M-LB was prepared by treating LB with Chelex 100 resin (50 mg/l; Bio-rad, Hercules, Ca) for 1 h at room temperature to remove heavy metal ions. The resin was removed by filtration and *N,N,N*’,N’-tetrakis (2-pyridylmethyl)-ethylenediamine (TPEN; Sigma) was added to a final concentration of 20 µM to further remove zinc. M-LB was replenished with divalent cations and metals by adding FeCl_3_, CaCl_2_, and MgCl_2_ to a final concentration of 1 µM, 1 µM, and 20 µM, respectively. Finally, 1 ml/l of trace elements SL-7 solution [46] lacking Zn was added. When necessary, cultures were supplemented with 200 µg/ml carbenicillin, 100 µg/ml ampicillin, 30 µg/ml gentamicin, or 50 µg/ml kanamycin to maintain plasmids.

### Generation of Mutant Strains

Unmarked in-frame deletion mutagenesis was used to create mutants by allelic exchange using a modification [47] of the previously described method by Horton et al. (1989) [48]. Briefly, mutant alleles were constructed using a two-step PCR protocol to create in-frame deletions of the DNA coding sequence corresponding to amino acids 28 to 138 (72%) of *zur* (PA5499), 39 to 285 (80%) of *znuA* (PA5498), 40 to 223 (80%) of *znuB* (PA5500), and 38 to 226 (70%) of *znuC* (PA5501). The oligonucleotide primers used to produce the mutant alleles added an XbaI site to the ends of the mutant *zur* allele, EcoRI sites to the end of *znuA*, and HindIII sites to the ends of *znuB*, and *znuC* mutant alleles. The DNA fragments were digested with the appropriate enzyme, purified from an agarose gel, and ligated into pEX18Ap, which had been previously digested with the same enzyme. This produced suicide plasmids pZur-suc, pZnuA-suc, pZnuB-suc, and pZnuC-suc.

To introduce the mutant alleles into *P. aeruginosa* strain PAO1, this strain was transformed with each suicide vector using the electroporation method described by Choi and Schweizer [49]. Single cross-over integrants were selected on carbenicillin and screened for sucrose sensitivity. Mutants were then selected by plating integrants on LB plus 6% sucrose to remove the vector from the chromosome. The resultant colonies were screened on LB containing carbenicillin to ensure carbenicillin resistance had been lost. Potential mutants were then screened by PCR and confirmed by DNA sequencing.

### Construction of *lacZ* Reporter Plasmids and β-Galactosidase (β-Gal) Assays

The chromosomal *znuA*’*-lacZ* transcriptional fusion reporter strains *P. aeruginosa* PAO1.*znuA*’*-lacZ* and *P. aeruginosa* PAO-Zur.*znuA*’*-lacZ* were constructed using the method described by Choi and Schweizer [50]. A 425 bp product beginning 387 bp upstream from the *znuA* translational start site was amplified by PCR using strain PAO1 chromosomal DNA as template and primers designed to add a PstI site at the 5’ and an HindIII site at the 3’ end. The fragment was digested with PstI and HindIII, purified from an agarose gel, and ligated into the pUC18-mini-Tn*7*T-Gm-*lacZ* plasmid, which had been previously digested with the same restriction enzymes. The resultant plasmid, p*znuA*’*-lacZ* was sequenced to confirm no mutations were introduced during cloning. The *znuA*’*-lacZ* fusion was inserted into the *attTn7* site on the *P. aeruginosa* strain PAO1 and strain PAO-Zur chromosomes as described elsewhere [49]. The gentamicin resistance cassette was excised using the pFLP2-encoded Flp recombinase, and the pFLP2 plasmid was cured via plating on 6% sucrose. A *zur*’*-lacZ* reporter plasmid was constructed by amplifying a 464 bp sequence containing the *zur* promoter (-307 to +164 relative to the *zur* translational start sight) by PCR using cosmid pMTP331 as a template. The primers were designed to add a PstI site at the 5’ end of the PCR product and an HindIII site at the 3’ end. The PCR fragment was digested with PstI and HindIII, purified from an agarose gel, and ligated into pUC18-mini-Tn*7*T-Gm-*lacZ* plasmid which had been previously digested with the same enzymes to produce p*zur*’*-lacZ*. Plasmids were sequenced to ensure that mutations were not introduced by PCR manipulations and the plasmids were introduced into the *attTn7* site on the *P. aeruginosa* strain PAO1 and PAO-Zur by electroporation as described above [49]. The gentamicin resistance cassette was excised from the chromosome using pFLP2 as described elsewhere [50].

To determine the effect of different zinc concentrations on *znuA* expression, cells from an overnight culture of *P. aeruginosa* strain PAO1.*znuA*’*-lacZ* and PAO-Zur.*znuA*’*-lacZ* were washed once in M-LB and used to inoculate 1 ml of fresh M-LB or M-LB, supplemented with different ZnCl_2_ concentrations, to an optical density at 660 nm (OD_660_) of 0.08. The cultures were grown for 3 h at 37°C with rotary aeration of ≥200 rpm and the β-Gal activity of each sample was assayed in duplicate. In some instances, PAO-Zur.*znuA*’*-lacZ* was transformed with pZur and β-gal assays were performed in M-LB supplemented with ZnCl_2_, carbenicillin 200 µg/ml, and 1% arabinose as indicated.

Data are reported in Miller units as the mean ± standard deviation (SD) of at least three separate experiments.

To determine the role of Zur on *zur* expression, cells from an overnight culture of *P. aeruginosa* strain PAO1.*zur*’*-lacZ* and PAO-Zur.*zur*’*-lacZ* were washed once in M-LB and used to inoculate 1 ml of fresh M-LB or M-LB, supplemented with different ZnCl_2_ concentrations, to an optical density 660 nm (OD_660_) of 0.08. The cultures were grown for 3 h at 37°C with rotary aeration of ≥200 rpm and the β-Gal activity of each sample was assayed in duplicate. Data are reported in Miller units as the mean ± standard deviation of at least three separate experiments.

### Construction of a Zur_PA_ Expression Plasmid

To construct a Zur_PA_ complementation plasmid, the oligonucleotide primers Zur_F and Zur_R (see Table S2) were used to amplify a 505 bp fragment containing the *zur* coding fragment from cosmid MTP331. The primers were designed to add a PciI site to the 5’ end and a HindIII site to the 3’ end of the PCR product. The PCR product was purified from an agarose gel, digested with NcoI and HindIII, and ligated into pHerd20T which had been digested with the same enzymes. The resulting plasmid, pZur, was sequenced to confirm that no mutations were introduced during DNA manipulation.

### Bioinformatics

The protein sequence of previously characterized Zur proteins found in *Escherichia coli*, *Salmonella* *typhimurium* , *Yersinia pestis*, and *Xanthomonas campestris* were obtained from the NCBI database. The protein sequence alignment of select Zur proteins was generated using ClustalW [51].

### RT-PCR

Total RNA was prepared from *P. aeruginosa* strain PAO1 mid-log phase cultures grown in LB using a Qiagen RNase Mini Kit. Contaminating DNA was removed by digestion with 10 U of RQ1 RNase-free DNase (Promega) for 2 h at 37°C. DNA-free total RNA was then used as a template for reverse transcriptase reactions using the Access RT-PCR Introductory System (Promega). The primers used in the RT-PCR reactions are listed in Table S2 and were in the following sets: A1, N1; N2, C1; C2, B1; B2, H1.

### Growth Kinetic Bioassays

The ability of strains PAO1, PAO-ZnA, PAO-ZnB, and PAO-ZnC to grow in zinc deplete conditions was determined by growth bioassays. Briefly, overnight cultures were diluted in LB supplemented with 0.5 mM EDTA to an OD_630_ of approximately 0.025. The cultures were grown at 37°C for 18 h with rotary aeration of ≥ 220 rpm. Growth was determined spectrophotometrically and results are presented as the mean of three independent biological replicates. Statistical significance of growth patterns was determined by the student T-test.

### Determination of Intracellular Zinc Concentrations

The intracellular concentration of zinc inside strain PAO1 and strain PAO-Zur was determined by inductively coupled plasma atomic emission spectroscopy (ICP-AES). Overnight cultures of strain PAO1 and PAO-ZnC were washed in LB and used to inoculate 5 ml LB to an OD_660_ of 0.05. The cultures were grown at 37°C with rotary aeration of ≥ 220 rpm for 2.5 h, then ZnCl_2_ was added to final concentration of 100 µM and the cultures were incubates as before for 3 h. The cells were pelleted by centrifugation at 5,000 x g for 5 min. at 4°C and then washed sequentially in 10 ml LB and 10 ml wash buffer (0.1 M lithium chloride, 0.2 mM ethylenediaminetetraacetic acid, and 0.1 mM ethylene glycol tetraacetic acid) to remove exogenous zinc. The cells were placed at 80°C overnight to dehydrate the cells, their dry weight was determined, and then incinerated at >800°C overnight. The samples were reconstituted in 2% nitric acid to a mass of10 g. The total zinc content was determined using (ICP-AES). The results of at least four replicates are reported as total zinc (mg) per gram of dry bacterial cell weight.

### Real Time RT-PCR

Total RNA was isolated from strains PAO1 and PAO-Zur grown in M-LB supplemented with 1 mM ZnCl_2_ to mid-log phase using a RNeasy Midi Kit (Qiagen) according to the manufacturer’s instructions. Contaminating DNA was removed using DNA-free (Ambion) Dnase Treatment and cDNA was generated using 2 µg total RNA, random primers, and Superscript III (Invitrogen) per manufacturer’s suggestions. The resultant cDNA was diluted 1:100 in nuclease free water and stored at -20°C until needed. Real time RT-PCR reactions were performed on a CFX96 real time system (Biorad) using 0.5 µl dilute cDNA as template, 200 nM final concentration of primers ZnuA_rt1 and ZnuA_rt2, or primer rplU_rt1 and rplU_rt2, 7.5 µl of PerfeCTa SYPR Green FastMix 2X mix (Quanta Biosciences), and ultrapure water up to a final volume of 25 µl. The ratio of *znuA* expression in strain PAO1 compared to strain PAO-Zur grown in high zinc concentrations was determined using the Pfaffl equation [52] using expression of *rplU* as a constant. The ratio was determined from the averages of at least three replicates from three independent biological replicates.

### Expression and Purification of Recombinant Zur_PA_

To construct a plasmid for expression of recombinant Zur_PA_, the oligonucleotide primers rZur_F and rZur_R (see Table S2) were used to amplify a 536 bp fragment containing the *zur* coding region with BsaI restriction sites added to the 5’ and 3’ ends. The PCR product was gel purified, digested with BsaI, and ligated into pASK-IBA6 (IBA GMH, Göttingen, Germany), which had been previously digested with the same enzyme. The resulting plasmid, prZur, containing a N-terminal Strep-tag II version of *P. aeruginosa* Zur (rZur_PA_), was introduced into *E. coli* strain DH5α by chemical transformation. A high concentration of prZur was purified from *E. coli* strain DH5α using a Qiagen Plasmid Midi kit, the plasmid was sequenced to insure no mutations were introduced during cloning, and the plasmid was transformed into chemically competent *E. coli* BL21(DE3).

For expression and purification of the rZur_PA_, overnight *E. coli* BL21(DE3) (prZur) cultures were used to inoculate 1 L of LB supplemented with ampicillin (100 µg/ml) contained in a 4 L growth flask. The cultures were grown at 37°C with rotary aeration ≥ 280 rpm until the OD_590_ reached 0.6, at which time anhydrotetracycline was added to a final concentration of 0.2 µg/ml and the cultures were incubated at 30°C with rotary aeration ≥ 280 rpm for 3 h. All subsequent steps were performed on ice or at 4°C. Cells were harvested by centrifugation at 5,000 x g for 5 min, and the cells were suspended in 10 ml lysis buffer (10 mM Tris-HCl, pH 8.0, 40 mM KCl, 10 mM MgCl_2_, 1 mM DTT, and 5% glycerol). Then the cells were lysed by sonication using a Branson Sonifer 450 (7 x 20 sec). The lysate was centrifuged at 5,000 x g for 15 min to remove the insoluble fraction and the aqueous lysate was passed through a 0.22 µM syringe filter apparatus. The lysate was applied to Strep-tactin gravity flow column (IBA GMH) per manufacturer’s instructions. Protein fractions were pooled and dialyzed in storage buffer (10 mM Tris-HCl, pH 8.0, 40 mM KCl, 10 mM MgCl_2_, 1 mM DTT, and 10% glycerol) and concentrated using an Amicon Ultra centrifugal filter. The protein concentration was determined by Bradford assays and the protein was stored at -70°C until used.

### Electrophoretic Mobility Shift Assays (EMSA)

The ability of Zur_PA_ to bind the *znuA* promoter was determined by EMSA essentially as described by Knoten et al. [53] Briefly, a 259 bp PCR fragment containing the *znuA* promoter (227 bp upstream from the GTG start codon to 32 bp downstream) was generated using strain PAO1 chromosomal DNA as a template and was labeled with [^32^P]ATP (PerkinElmer, Wellesley, MA) by using T4 polynucleotide kinase (Invitrogen, Carlsbad, CA). The binding assays were carried out in binding buffer containing 10 mM Tris-HCl, pH 8.0, 40 mM KCl, 10 mM MgCl_2_, 1 mM DTT, and 5% glycerol and 1 µM ZnCl_2_. Each reaction mixture contained 0.5 µg of salmon sperm DNA, 10^4^ cpm radiolabeled DNA, and 0 to 800 ng of protein. Where indicated, 100x excess specific competitor (unlabeled *znuA* promoter), 100x excess non-specific competitor (unlabeled internal fragment of *znuC*), or 5 µM TPEN was added to the binding reaction. Reaction mixtures were incubated at room temperature for 30 min and separated by electrophoresis at 4°C on a native 7.5% polyacrylamide gel supplemented with 2.5% glycerol in 1X Tris-borate buffer. Radiolabeled bands were visualized by autoradiography after the X-ray film had been exposed for 18 h.

## Supporting Information

Figure S1 **Growth Kinetics of *P. aeruginosa* strains PAO1, PAO-ZnA, PAO-ZnB, and PAO-ZnC grown in LB medium.** Overnight cultures of each strain were diluted in fresh LB media to an OD_630_ of 0.005 and the cultures were incubated at 37°C with rotary aeration ≥ 220 rpm. Growth was monitored spectrophotometrically for 6 hours. The results presented are the mean of three independent experiments.
(TIF)

Table S1 **List of bacterial strains and plasmids used in this study.**
(DOCX)

Table S2 **List of primers used in this study.**
(DOCX)
