# Correction: The Transcriptional Regulator Np20 Is the Zinc Uptake Regulator in *Pseudomonas aeruginosa*

**DOI:** 10.1371/annotation/40be2093-f3d0-41ca-a8d7-02fecd051942

**Published:** 2013-10-15

**Authors:** Matthew L. Ellison, John M. Farrow, Whitney Parrish, Allison S. Danell, Everett C. Pesci

The name of the second author was given incorrectly. The correct name is: John M. Farrow III. The correct citation is: Ellison ML, Farrow JM III, Parrish W, Danell AS, Pesci EC (2013) The Transcriptional Regulator Np20 Is the Zinc Uptake Regulator in *Pseudomonas aeruginosa*. PLoS ONE 8(9): e75389. doi:10.1371/journal.pone.0075389.

In addition, multiple funding organizations and a grant were incorrectly omitted from the Funding Statement. The Funding Statement should read: "This work was supported by a research grant from the National Institute of Allergy and Infectious Disease to ECP (grant AI076272) and a new investigator award from the Kentucky Biomedical Research Infrastructure Network and INBRE to MLE (funded by grant 8P20GM103436-12 from the National Institute of General Medicine). The funders had no role in study design, data collection and analysis, decision to publish, or preparation of the manuscript." 

