# Supplementary figures and images for: The Transcriptional Regulator Np20 Is the Zinc Uptake Regulator in Pseudomonas aeruginosa
Source: PLoS One. 2013 Sep 23;8(9):e75389. doi: 10.1371/journal.pone.0075389 (PMC3781045; doi:10.1371/journal.pone.0075389)

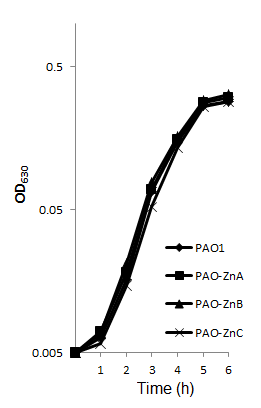

Supplement: Figure S1 — Growth Kinetics of P. aeruginosa strains PAO1, PAO-ZnA, PAO-ZnB, and PAO-ZnC grown in LB medium. Overnight cultures of each strain were diluted in fresh LB media to an OD630 of 0.005 and the cultures were incubated at 37°C with rotary aeration ≥ 220 rpm. Growth was monitored spectrophotometrically for 6 hours. The results presented are the mean of three independent experiments. (TIF) [file pone.0075389.s001.tif]
