# Supplementary material for: The Transcriptional Regulator Np20 Is the Zinc Uptake Regulator in Pseudomonas aeruginosa
Source: PLoS One. 2013 Sep 23;8(9):e75389. doi: 10.1371/journal.pone.0075389 (PMC3781045; doi:10.1371/journal.pone.0075389)
Supplement: Table S1 — List of bacterial strains and plasmids used in this study. (DOCX) [file pone.0075389.s002.docx]

**Table S1.** Bacterial strains and plasmids used in this study.

| **Strain or plasmid** | **Relevant genotype or phenotype** | | **Reference** |
| --- | --- | --- | --- |
| ***E. coli* strains** |  | |  |
| **DH5α** | F- φ80*lac*ZΔM15 Δ(*lac*ZYA-*arg*F) U169 *rec*A1 *end*A1 *hsd*R17(r_k_^-^, m_k_^+^) *pho*A *sup*E44 *thi*-1 *gyr*A96 *rel*A1 λ- | | Invitrogen |
| **BL21(DE3)** | F^–^ *ompT gal dcm lon hsd*S_B_(r_B_^-^ m_B_^-^) λ(DE3 [*lacI lac*UV5-T7 gene 1 ind1 *sam7 nin5*]) | Novagen | |
| ***P. aeruginosa* strains** |  |  | |
| **PAO1** | Wild-type *P. aeruginosa* strain | [[51](#_ENREF_51)] | |
| **PAO-Zur** | *zur* deletion mutant derived from strain PAO1 | This study | |
| **PAO-ZnA** | *znuA* deletion mutant derived from strain PAO1 | This study | |
| **PAO-ZnB** | *znuB* deletion mutant derived from strain PAO1 | This study | |
| **PAO-ZnC** | *znuC* deletion mutant derived from strain PAO1 | This study | |
| **PAO1.*znuA’-lacZ*** | Wild type strain with a chromosomal *znuA’-lacZ* transcriptional fusion | This study | |
| **PAO-Zur.*znuA’-lacZ*** | *zur* deletion strain with a chromosomal *znuA’-lacZ* transcriptional fusion | This study | |
| **PAO1.*zur’-lacZ*** | Wild type strain with a chromosomal *zur’-lacZ* transcriptional fusion | This study | |
| **PAO-Zur.*zur’-lacZ*** | *zur* deletion strain with a chromosomal *zur’-lacZ* transcriptional fusion | This study | |
| **Plasmids** |  |  | |
| **pMTP331** | Cosmid vector containing *zur* and *znuABC* genes, Tet^r^ | [[52](#_ENREF_52)] | |
| **pEX18Ap** | Suicide vector for *P. aeruginosa*; Amp^r^ | [[53](#_ENREF_53)] | |
| **pΔZur-suc** | *zur* deletion suicide vector; Amp^r^ | This study | |
| **pΔZnuA-suc** | *znuA* deletion suicide vector; Amp^r^ | This study | |
| **pΔZnuB-suc** | *znuB* deletion suicide vector; Amp^r^ | This study | |
| **pΔZnuC-suc** | *znuC* deletion suicide vector; Amp^r^ | This study | |
| **pZur-lacZ** | *zur’-lacZ* transcriptional chromosomal fusion vector; Amp^r^ | This study | |
| **pZnuA-lacZ** | *znuA’-lacZ* transcriptional chromosomal fusion vector; Amp^r^ | This study | |
| **pUC18-mini-Tn7T-Gm-**  **lacZ** | Gm^r^ on mini-Tn7T; *lacZ* transcriptional fusion vector | [[46](#_ENREF_46)] | |
| **pTNS2** | Transposition helper plasmid; Amp^r^ | [[46](#_ENREF_46)] | |
| **pFLP2** | Source of the Flp recombinase used to excise the FRT-flanked Gm^r^ | [[46](#_ENREF_46)] | |
| **pASK-IBA6** | Strep-tagged protein expression vector; Amp^r^ | IBA Lifesciences | |
| **prZur** | Strep-tagged Zur expression vector on pASK-IBA6, Amp^r^ | This study | |
| **pHerd20T** | Expression vector under inducible pBAD promoter, Amp^r^ | [[54](#_ENREF_54)] | |
| **pZur** | Zur expression plasmid on pHerd20T, Amp^r^ | This study | |

Abbreviations: Gm^r^, gentamicin resistance; Amp^r^, ampicillin resistance; Km^r^, kanamycin resistance; Tet^r^, tetracyline resistance.
