# Supplementary material for: The Transcriptional Regulator Np20 Is the Zinc Uptake Regulator in Pseudomonas aeruginosa
Source: PLoS One. 2013 Sep 23;8(9):e75389. doi: 10.1371/journal.pone.0075389 (PMC3781045; doi:10.1371/journal.pone.0075389)
Supplement: Table S2 — List of primers used in this study. (DOCX) [file pone.0075389.s003.docx]

| **TABLE S2**. Primers used in this study. | |
| --- | --- |
| **Primer** | **Sequence (5’ to 3’)** |
| Zur_O1.2 | AAAATCTAGACGGGTTTTCAGAGCTTTTCC |
| Zur_I1 | CTCGACGGTCTGGCTTTCGCACTGGCTGTGGTCATG |
| Zur_I2 | CATGACCACAGCCAGTGCGAAAGCCAGACCGTCGAG |
| Zur_O2.2 | AAAATCTAGAGACAGGGTATCGCCGAAATA |
| ZnuA_O1 | AAA AAGAATTCG GAG TTT CTG CGG CAT GTA G |
| ZnuA_I1 | AAACCGCTGCAACTGGTCGACGCTAACGGCTATGAGAATCTT |
| ZnuA_I2 | AAGATTCTCATAGCCGTTAGCGTCGACCAGTTGCAGCGGTTT |
| ZnuA_O2 | AAA AAGAATTCG TGA TCA GCA GGC TCA GGT T |
| ZnuB_O1 | AAAAAAAGCTTGGGCCAGTTCCTCATCTG |
| ZnuB_I1 | ACTCAGCAGGAACAGGCCGGACAGGGTATCGCCGAAATA |
| ZnuB_I2 | TATTTCGGCGATACCCTGTCCGGCCTGTTCCTGCTGAGT |
| ZnuB_O2 | AAAAAAGCTTAACCGAGCATTTTACCTTGCT |
| ZnuC_O1 | AAAAAAGCTTCCTCTTCCTCGAAATTGACG |
| ZnuC_I1 | GTCGTGATGGTGGTGGTAGATGATCAGGGTGACGATCTG |
| ZnuC_I2 | CAGATCGTCACCCTGATCATCTACCACCACCATCACGAC |
| ZnuC_O2 | AAAAAAGCTTATCATTCCTGCGGGTTCTC |
| A1 | AGCGGTTTGATGCTGGTC |
| N1 | TCTGCCAGACCAGTTCCAG |
| N2 | AGGGCCAGTTCCTCATCTG |
| C1 | TCGACATCGCTCAGCACTG |
| C2 | ATCTACCACCACCATCACGA |
| B1 | ACAGGGTATCGCCGAAATAG |
| B2 | TGTCCTGGTTCAAGGATACC |
| H1 | TTTCCTGCAGTTCGGCATAG |
| ZnuA_RT1 | GCGGGCTCGACGGGAAACTC |
| ZnuA_RT2 | ACCGCGAACACGCCGGTATG |
| RplU_RT1 | GGTGGCAAGCAGCACAAAGTCACCG |
| RplU_RT2 | GCGGACCTTGTCGTGACGGCCGTGG |
| rZur_F | CGCGGTCTCAGCGCATGTACAAGATTGCGCCCAAGACCC |
| rZur_R | GCGCGGTCTCATATCAGGCGTCCTTCTGGTCCCG |
| Zur_HerdF | AAAAACATGTACAAGATTGCGCCCAA |
| Zur_HerdR | AAAAAAGCTTTCAGGCGTCCTTCTGGTC |
| Zur_LacZ_F | AAACTGCAGGGGCCGATCCAGTAGAACAAT |
| Zur_LacZ_R | AAAAAGCTTGCA CTG GCT GTG GTC ATG |
| ZnuA_LacZ_F | AAAACTGCAGGTTGTTGCAGCCGACGAACGCGCG |
| ZnuA_LacZ_R | AAAAAAGCTTGAAGCAGGTGGACAGCAGGGCAAA |
| ZnuA_EMSA_F | TGCTGACGCACTGGCTGTGG |
| ZnuA_EMSA_R | CGGCGAAGCCAGCAGGAACA |
| ZnuC_EMSA_F | GGCAAGACCACCCTGGTA |
| ZnuC_EMSA_R | GGTACCAGGCGGAGGAAG |
